# Hydrothermal Synthesis of ZnO@MnO_2_‑Montmorillonite Nanocomposites: Influence of Molarity on Structural, Optical, and Photocatalytic Performance toward Ciprofloxacin Degradation under Variable Conditions

**DOI:** 10.1021/acsomega.5c06454

**Published:** 2025-09-19

**Authors:** Elisabethe Bezerra, Williams A. Santos Albuquerque, Adilson J. Neres Filho, Alexsandro Lins, Ricardo Barbosa, Luciano C. Almeida, Santiago Medina-Carrasco, Maria del Mar Orta Cuevas, Josy A. Osajima, Pollyana Trigueiro, Ramón Raudel Peña Garcia

**Affiliations:** 1 Programa de Pós-Graduação em Engenharia Física, Unidade Acadêmica do Cabo de Santo Agostinho, Universidade Federal Rural de Pernambuco,Cabo de Santo Agostinho,PE 54518-430, Brazil; 2 Programa de Pós-Graduação em Ciência de Materiais, 28116 Universidade Federal de Pernambuco,Recife,PE 50740-560, Brazil; 3 Departamento de Engenharia Química, 28116 Universidade Federal de Pernambuco, Recife,PE 50740-590, Brazil; 4 SGI Laboratorio de Rayos X - Centro de Investigación, Tecnología e Innovación de la Universidad de Sevilla (CITIUS), Sevilla 41012, España; 5 Departamento de Química Analítica, Facultad de Farmacia, 16778 Universidad de Sevilla, Sevilla 41012, España; 6 Laboratório Interdisciplinar de Materiais Avançados (LIMAV), Centro de Tecnologia, Teresina,PI 64049-550,Brazil

## Abstract

In this work, a series of ZnO@MnO_2_-montmorillonite nanocomposites was successfully synthesized via a hydrothermal route using NaOH solutions at varying molarities (3–9 M) and evaluated for their structural, optical, morphological, and photocatalytic properties, with specific application in ciprofloxacin degradation. Structural investigation confirmed the coexistence of pure ZnO (hexagonal), MnO_2_ (tetragonal), and montmorillonite phases and showed molarity-dependent variations in crystallite size, from 34 nm (ZMM5) to 7 nm (ZMM9), and dislocation density, which ranged from 0.0009 to 0.0180 nm^–2^. Fourier-transform infrared spectroscopy (FTIR) spectra evidenced the progressive occupation of montmorillonite OH sites by metal oxides. At the same time, photoluminescence deconvolution revealed a shift in dominant intrinsic defects: ZMM3 exhibited 49% Zn vacancies (*V*_Zn_), whereas ZMM7 and ZMM9 showed higher concentrations of oxygen vacancies (*V*_O_^+^ up to 40.7%). The band gap narrowed from 3.294 eV (ZMM3) to 2.945 eV (ZMM9), indicating an increase in defect states and enhanced light absorption. Textural analysis revealed that ZMM3 had the highest surface area (35.1 m^2^ g^–1^) and microporosity, whereas samples synthesized at higher molarities exhibited mesoporous structures with reduced surface areas (∼18–22 m^2^ g^–1^). Photocatalytic tests under ultraviolet (UV) irradiation for 120 min showed excellent degradation performance for all nanocomposites: ZMM3 (60.1%), ZMM5 (61.0%), ZMM7 (59.7%), and ZMM9 (59.5%), indicating robust activity independent of structural variations. Operational parameter studies confirmed that ZMM5 maintained high activity across different catalyst dosages (25–100 mg (47–61%)), pollutant concentrations (10–30 mg L^–1^ (45–61%)), and pH levels (3–9 (48–61%)), achieving degradation rates between 45 and 61%. Scavenger tests confirmed hydroxyl radicals (•OH) and holes (h^+^) as the main active species. Reusability tests revealed a 28% drop after three cycles (from 59 to 31%), while X-ray diffraction (XRD) confirmed structural stability with no formation of secondary phases. These results demonstrate the robustness, tunability, and applicability of the ZnO@MnO_2_-montmorillonite system for real-world water treatment under variable conditions.

## Graphical abstract

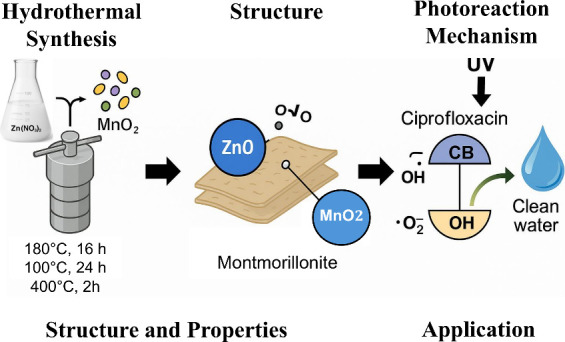

## 1 Introduction

Zinc oxide (ZnO) is a widely recognized semiconductor known for its utility in catalytic processes. Its desirable properties, such as good thermal and chemical stability, affordability, ease of synthesis, and biocompatibility, make it an attractive option for various applications.^1–5^ However, the practical utility of heterogeneous photocatalysis processes of ZnO is hindered by the rapid recombination of electron–hole pairs, which negatively impacts their photocatalytic efficiency. Furthermore, ZnO tends to exhibit poor quantum efficiency, insufficient reduction potential, and limited electron mobility.^6^ To enhance the performance of ZnO, one promising strategy involves doping or combining it with other semiconductor oxides, such as CuO, NiO, CdS, CeO_2_, TiO_2_, or MnO_2_. This approach creates heterostructures that foster improved charge separation. MnO_2_ is notable for its abundance, low cost, narrow energy band, strong oxidative properties, and nontoxic nature.^7^ MnO_2_ functions as an electron scavenger, effectively inhibiting recombination and prolonging the lifespan of charge carriers due to its advantageous electronic structure and redox properties.^8^ The charge transfer mechanisms in ZnO-based heterostructures can be influenced by several factors, including band gap adjustment, interface and surface defects, surface state density, the wavelength of irradiated light, and reaction conditions such as pH, temperature, and sacrificial reagents.^9–13^ Understanding and controlling these variables is essential for optimizing the efficiency of ZnO-based photocatalysts in diverse applications, particularly in photocatalytic processes.

Incorporating semiconductor nanoparticles onto solid supports, such as clay minerals, can enhance the degradation of pollutant molecules under UV or visible light while allowing for multiple reuse cycles with minimal loss of efficiency. This approach positions nanocomposites based on clay minerals as environmentally friendly and economically viable options for the remediation of contaminated water. Several studies have reported the beneficial effects of depositing different semiconductors onto various clay minerals. For example, Mahy et al.^14^ produced nanocomposites using ZnO or TiO_2_ deposited on a natural smectite clay synthesized by the sol–gel method. They found that these materials exhibited high photocatalytic activity, achieving 90% degradation of p-nitrophenol under UVA irradiation over 8 h for the ZnO/Cu^2+^ composites. Liu et al.^15^ prepared ternary nanocomposites consisting of Bi_2_MoO_6_/g-C_3_N_4_/kaolinite using the solvothermal method and applied them to the photodegradation of tetracycline under visible light. They concluded that the kaolinite-based samples demonstrated superior performance, achieving over 90% drug removal compared to the bare materials. Kharouf et al.^16^ developed a TiO_2_/kaolin composite for the degradation of phenazopyridine, reporting that the kaolin-based composite was able to remove up to 90% of the drug within 60 min at a pH of 8.7. The authors emphasized the stability of the composite after four reuse cycles. Fatimah et al.^17^ synthesized NiO/montmorillonite using a hydrothermal method for the removal of tetracycline. The study found an efficiency of up to 94.2% under UV light and 75.5% under visible light exposure, with good stability observed after five cycles of catalyst reuse.

The combination of ZnO and MnO_2_ supported on montmorillonite clay mineral shows significant potential for degrading emerging contaminants, particularly ciprofloxacin, an antibiotic frequently found in hospital and urban wastewater. The heterojunction formed between ZnO and MnO_2_ can enhance charge separation and light absorption, while montmorillonite aids in the adsorption of ciprofloxacin onto the catalyst surface, thereby facilitating its photodegradation mechanism. Montmorillonite, a typical 2:1 type clay mineral from the smectite group, is known for its high surface area, ion exchange capacity, and excellent dispersibility in aqueous media. These properties facilitate the incorporation of nanoparticles, preventing agglomeration and promoting effective contact between the photocatalyst and target pollutants, thereby accelerating the reaction rate.^18–20^ Additionally, immobilizing these nanoparticles on inorganic supports like clay minerals provides a sustainable method to enhance the stability, dispersion, functionality, and reusability of the catalysts. Montmorillonite is especially effective due to its reactive surface area, expandable layered structure, and adsorbent properties, which enhance the uniform distribution of photocatalytic nanoparticles within its structure.

In this context, developing hybrid nanocomposites made of ZnO/MnO_2_ incorporated onto montmorillonite matrix represents a promising solution for the efficient degradation of Ciprofloxacin (CIP) in aqueous environments. Ciprofloxacin is a fluoroquinolone antibiotic widely used in human and veterinary medicine as well as aquaculture, and is frequently detected in urban, hospital, and industrial wastewater.^21–26^ Unmetabolized residues of this antibiotic contribute to soil and water pollution. These residues can accumulate in aquatic organisms and food crops, leading to human exposure, toxicity, and the development of bacterial resistance. The environmental persistence of ciprofloxacin and its potential to promote microbial resistance highlight the urgent need for advanced materials and technologies to decrease its prevalence.^27^ This study introduces a novel approach by synthesizing nanocomposites composed of zinc oxide and manganese dioxide nanoparticles supported on a montmorillonite matrix. The synthesis will be conducted using a hydrothermal method with varying sodium hydroxide concentrations. Although recent progress has been made in developing new nanostructures, the fabrication of ternary ZnO@MnO_2_-montmorillonite composites has not been previously documented. This research will systematically investigate the structural, morphological, optical, and photocatalytic properties of the synthesized material, with a focus on the efficient removal of ciprofloxacin under ultraviolet light irradiation.

## 2 Experimental Procedure

### 2.1 Materials

Zinc Nitrate Zn­(NO_3_)_2_·6H_2_O (99.5% purity) and Manganese­(IV) Oxide (MnO_2_, 99.0% purity) are purchased from Sigma-Aldrich. Montmorillonite natural clay mineral, is from San Juan Province, Argentina. Distilled water was used as a solvent for washing, ethanol for washing, and NaOH (0.1 M) solution was used for pH adjustment.

### 2.2 Hydrothermal Synthesis Procedure

Using the hydrothermal method, 1.0 g of montmorillonite clay mineral and 0.03718 g of manganese oxide were dispersed in 25 mL of distilled water. The mixture was stirred for 30 min. Separately, 3.718 g of zinc nitrate was dissolved in 25 mL of distilled water until a homogeneous solution was obtained. This zinc precursor solution was gradually combined with the clay mineral and manganese dispersion, and the mixture was stirred continuously for an additional 30 min. To adjust the pH, different concentrations of NaOH (3 M, 5 M, 7 M, and 9 M) were added while maintaining constant agitation. The mixture was then transferred to a Teflon cup and placed in a hydrothermal reactor. The system was heated in a muffle furnace at 180 °C for 16 h. The resulting solid was separated by centrifugation and washed five times with distilled water and twice with ethanol to remove any residues. Finally, the solid was dried at 100 °C for 24 h and subsequently calcined at 400 °C for 2 h. The samples obtained in this process were labeled ZMM3, ZMM5, ZMM7, and ZMM9, according to the concentration of NaOH, and they contain montmorillonite functionalized with manganese oxide and zinc oxide. The hydrothermal route enables precise control over nanoparticle size, morphology, and agglomeration. Additionally, this method facilitates the synthesis of compounds with well-defined and complex compositions and structures. The use of a closed system minimizes material losses and operates at lower temperatures compared to alternative synthesis methods, thereby reducing both costs and energy consumption. In this synthetic approach, parameters such as the ratio of starting materials, the pH of the medium reaction, and the temperature and reaction time can be finely tuned.^28^ An alkaline environment enhances the dispersion and integration of semiconductor nanoparticles, thereby improving the availability of active sites and facilitating electron transfer between the clay mineral structure and the metal oxide nanoparticles.^29^

### 2.3 Characterization Techniques

The structural and phase characteristics of the synthesized materials were examined using X-ray diffraction (XRD) with a Bruker D8 Advance system and Cu Kα radiation (λ = 1.5406 Å). Fourier-transform infrared spectroscopy (FTIR) was carried out using a Shimadzu IRXross spectrometer equipped with an ATR module. Each spectrum was acquired with 45 scans at a resolution of 4 cm^–^^1^, covering the range from 4000 to 400 cm^–^^1^. Surface morphology and microstructural details were assessed by scanning electron microscopy (SEM) using a TESCAN MIRA3, enabling high-resolution analysis of particle shape, size distribution, and homogeneity. Nitrogen adsorption–desorption measurements at 77 K were used to evaluate textural properties. The data were processed with NOVAWIN2 software to calculate specific surface area (BET method), pore volume, and pore size distribution (NLDFT or BJH/DFT models). Diffuse reflectance spectra were acquired using a Shimadzu UV-2700 UV–Vis spectrophotometer with an integrating sphere. Optical band gaps were estimated through Tauc plots based on the Kubelka–Munk function. Photoluminescence (PL) spectra at room temperature were obtained with a Horiba-Jobin Yvon Fluorolog-3 spectrofluorometer using 340 nm excitation. Spectral deconvolution was applied to analyze defect-related emissions and to gain insights into the electronic structure.

## 3 Results and Discussion

### 3.1 Structural Investigation by XRD and FTIR of the Nanocomposite

Figure 1a displays the X-ray diffraction (XRD) patterns of the ZnO@MnO_2_-Montmorillonite nanocomposite synthesized under different molar concentrations of sodium hydroxide (ZMM3, ZMM5, ZMM7, and ZMM9 samples). In addition, the diffractograms of the starting materials are highlighted in the Supporting Information (Figure S1).

**Figure 1:**
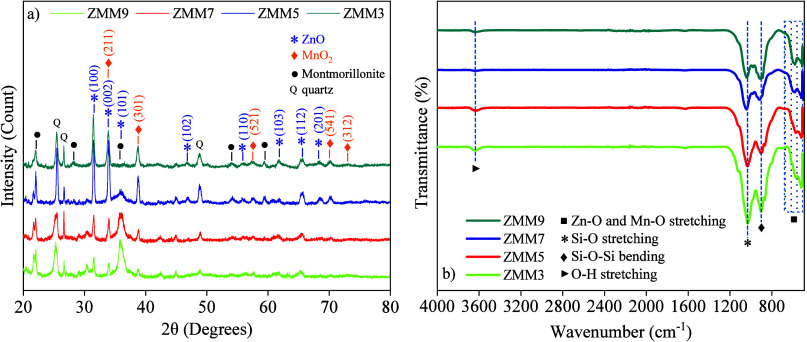
(a) XRD analysis and (b) FTIR measurements for the ZnO@MnO_2_-montmorillonite nanocomposite.

The analysis of Figure 1a indicates the coexistence of three well-defined crystalline phases, ZnO (hexagonal, JCPDS 36-1451), MnO_2_ (tetragonal, JCPDS 44-0141), and montmorillonite (JCPDS 03-0015), confirming the efficient formation of the hybrid structure. The results indicate that Zn^2^^+^ or Mn^2^^+^ cations do not substitute for any cations in the tetrahedral or octahedral sites of the montmorillonite structure. This suggests that the formation of ZnO and MnO_2_ phases occurs in situ. Furthermore, the interaction between the nanoparticles and the clay mineral structure takes place primarily on both the internal and external surfaces.^30^ In this context, the diffraction patterns exhibit characteristic peaks for each phase, with no detectable secondary phases within the instrument’s resolution, suggesting high phase purity and indicating the selectivity of the synthesis method employed. For the ZnO phase, the peaks at 31.41° (100); 33.90° (002); 36.13° (101); 46.71° (102); 55.82° (110); 62.01° (103); 68.18° (112); and 69.89° (201) are consistent with the hexagonal structure (space group *P*6_3_ *mc*), where the (100) plane exhibits the highest intensity, indicating a preferential crystallographic orientation along this direction.

The absence of residual peaks, such as Zn­(OH)_2_, further confirms the effectiveness of the calcination process in yielding phase-pure ZnO. The MnO_2_ phase exhibits reflections at 34.00° (211); 39.98° (301); 58.72° (521); 70.20° (541); 73.79° (312), consistent with a tetragonal structure (space group *P*4_2_/*mnm*). The oxidative stability of MnO_2_ is evidenced by the absence of reduced phases such as Mn_3_O_4_, highlighting the effective kinetic control achieved during the synthesis process.

On the other hand, montmorillonite exhibits diffraction peaks at 21.96° (110), 26.46° (quartz), 27.50° (quartz), 28.77° (004), 53.90° (201), and 59.98° (060). The presence of these planes provides structural support to the nanocomposite, serving as a matrix for the dispersion of ZnO and MnO_2_ nanoparticles, as further corroborated by the proximity or overlap of peaks with the montmorillonite matrix. This interfacial interaction, mediated by hydroxyl bonds or oxygen bridges, stabilizes the hybrid structure and enhances electronic properties, such as charge transfer between the phases. A careful inspection of Figure 1a reveals the influence of NaOH concentration on the structural parameters of the nanocomposite. The variation in sodium hydroxide concentration during the synthesis of the ZnO@MnO_2_-Montmorillonite nanocomposite acts as a key factor in modulating the chemical environment and, consequently, in driving structural reorganization at the atomic and nanometric scales. Considering that the majority phase aligns with ZnO in the nanocomposite, the lattice constants (*a* and *c*), bond length (*L*), dislocation density (δ), and crystallite size (*D*) were estimated for this phase, using the corresponding equations reported by Soares et al.^31,32^ and Castro-Lopes et al.^33^ for the ZnO crystal structure.

Table 1 presents the structural parameters calculated from the XRD patterns for the ZnO@MnO_2_-Montmorillonite nanocomposite. An in-depth analysis of the structural parameters calculated from the XRD patterns for the ZMM3, ZMM5, ZMM7, and ZMM9 samples reveals how varying the molarity using NaOH modulates the balance between nucleation, growth, defects, and crystal organization in the ZnO@MnO_2_-Montmorillonite nanocomposite. For the ZMM3 sample (3 M), the moderate pH generates low OH^–^ supersaturation in the bulk reaction medium, yet montmorillonite’s hydroxyl-rich lamellar surfaces act as local reservoirs of these ions and promote heterogeneous nucleation along the clay mineral sheets.

**Table 1:** Structural Parameter Obtained for the ZnO@MnO_2_-Montmorillonite Nanocomposite

	ZnO@MnO_2_-montmorillonite			
parameters	ZMM3	ZMM5	ZMM7	ZMM9
*a* (Å)	3.2825(1)	3.2770(1)	3.2785(2)	3.2780(1)
*c* (Å)	5.2850(2)	5.2755(2)	5.2740(1)	5.2746(2)
bond length *L* (Å)	2.0008	1.9974	1.9978	1.9977
dislocation density δ (nm^–2^)	0.0014	0.0009	0.0141	0.0180
crystallite size *D* (nm)	27	34	8	7

This localized concentration of OH^–^ reduces the activation energy for nucleus formation but, because of the limited overall availability of hydroxide, promotes surface-controlled, anisotropic growth regime. As a result, the composite exhibits expanded lattice parameters (*a* = 3.2825 Å; *c* = 5.2850 Å), compared to those reported in the literature for other ZnO-based composites,^34,35^ a high value of Zn–O bonds (2.0008 Å), intermediate crystallite size (27 nm), and an elevated dislocation density (0.0014 nm^–2^). These characteristics reflect elevated surface energy, compensatory oxygen vacancies, and lattice strains arising from partially occupied Na^+^ exchange sites, collectively highlighting montmorillonite’s dual function as both an ionic mediator and a structural scaffold.

By increasing the NaOH molarity to 5 M (ZMM5), the elevated OH^–^ concentration consistently raises the reaction pH while also enhancing montmorillonite’s cation-exchange activity, releasing Na^+^ from structural sites and displacing the interlayer water. This dual modulation of the ionic environment accelerates Zn^2^^+^ and MnO_4_^–^ hydrolysis and promotes controlled condensation of hydroxides into stable nuclei, all the while reducing oxygen vacancies through tighter ionic packing. Consequently, the hexagonal lattice contracts significantly (*a* = 3.2770 Å; *c* = 5.2755 Å) and Zn–O bonds shorten (*L* = 1.9974 Å), establishing a diffusion-controlled growth regime that produces larger crystallites (34 nm) and minimizes dislocation density (0.0009 nm^–2^), characteristics of superior crystallinity. Crystallinity can significantly enhance a material’s photocatalytic activity by improving light absorption. Additionally, it facilitates the mobility of photogenerated charges, enabling their efficient migration to the surface and subsequent participation in redox reactions.

For the ZMM7 sample, the supersaturation exceeds the diffusion-controlled regime, and montmorillonite, already saturated with Na^+^, acts predominantly as an ionic buffer: its charged lamellae disperse nascent nuclei throughout the medium yet impede their lateral aggregation into larger crystallites. Consequently, despite the maintenance of a compact hexagonal lattice (*c* = 5.2740 Å) and persistently short Zn–O bonds (≈ 1.9978 Å), the unit-cell parameter (*a*) experiences a slight expansion to 3.2785 Å as internal lattice tensions build. This rapid, heterogeneous nucleation dramatically reduces the *D* value to 8 nm, while the competition among numerous nuclei and the clay mineral-mediated interfacial stresses amplify linear defects, driving the δ up to 0.0141 nm^–2^. Thus, the interplay between excessive OH^–^ supersaturation, Na^+^-surface interactions, and montmorillonite’s lamellar structure causes a shift from ordered growth to defect-rich, nanometric domains.

Finally, at 9 M (ZMM9), the system reaches a critical ionic saturation where the excessive OH^–^ concentration causes nearly instantaneous nucleation across the ZnO@MnO_2_-Montmorillonite interface, while the montmorillonite, fully loaded with interlayer Na^+^, can no longer mediate ion diffusion or relieve lattice stress. This leads to an apparent stability in the lattice constants (*a* = 3.2780 Å; *c* = 5.2746 Å) and persistently short Zn–O bonds (1.9977 Å), yet the overwhelming number of nuclei stifles further growth, yielding minimal crystallite sizes (7 nm) and maximal dislocation density (0.0180 nm^–^^2^). In essence, montmorillonite acts not merely as an inert support but as a dynamic ionic reservoir and diffusion barrier, modulating local pH microenvironments, Na^+^/OH^–^/H_2_O exchange, and interfacial stress, which, in concert with NaOH molarity, finely tunes the balance between defects, crystalline order, and morphology, ultimately dictating the composite’s active-site density and its photocatalytic and electrochemical performance. Fourier transform infrared (FTIR) spectra were also obtained for the ZnO@MnO_2_-Montmorillonite nanocomposite, aiming to analyze its chemical and molecular structure. The measurements were performed in the spectral range from 400 cm^–^^1^ to 4000 cm^–^^1^, as shown in Figure 1b. The discrete band observed at ∼3640 cm^–^^1^ corresponds to the O–H stretching vibration of hydroxyl groups of the montmorillonite structure, indicating the presence of internal hydrogen bonds.^36^ The intense absorption peak located at ∼1028 cm^–^^1^ corresponds to the stretching vibration of the Si–O bond within the montmorillonite,^37,38^ while the peak located at ∼906 cm^–^^1^ can be attributed to the bending vibration of the Si–O–Si.^39^ The presence of these bands in the spectra suggests that the clay mineral structure is present in the ZnO@MnO_2_-Montmorillonite nanocomposite. On the other hand, increasing the molarity during the synthesis step can promote a greater availability of OH^–^ ions in the reaction medium, which favors the nucleation and growth of metal oxides.^12,33^ This can cause the progressive deposition of ZnO and MnO_2_ in the montmorillonite layers, which can modify the presence of hydroxyl and siloxyl groups and decrease the apparent intensity of the bands associated with these groups. Finally, the absorption bands located at ∼567 cm^–^^1^ and ∼490 cm^–^^1^ can be attributed to the stretching vibration of the Mn–O and Zn–O bonds, respectively, evidencing the presence and interaction of metal oxides with the montmorillonite matrix.^40,41^ Analyzing the spectra in detail, it is noted that for the ZMM3 sample (3 M) the spectrum is marked by the inherent montmorillonite O–H stretching band at ∼3640 cm^–^^1^, broad stretches at ∼1028 cm^–^^1^ of Si–O band and strong Si–O–Si bonds stretches near ∼906 cm^–^^1^ with not perfectly define band of the metal oxides.^37^ Specifically, only a weak Zn–O band (wurtzite ZnO) appears near ∼490 cm^–^^1^ and Mn–O mode (MnO_2_) around ∼567 cm^–^^1^. As the NaOH concentration increases to 5 and 7 M (ZMM5 and ZMM7), hydrolysis of the Zn precursor becomes more complete, yielding more defined ZnO and MnO_2_ bands. In addition, the O–H stretching band of montmorillonite steadily diminishes in intensity as the base level rises, indicating the progressive coverage of the clay mineral’s OH sites by ZnO/MnO_2_ and likely Zn^2^^+^/(Mn^2^^+^) exchange with the interlayer cations.^37^ The Si–O–Si stretching subtly shifts and broadens at higher pH, indicating partial disruption or reorganization of the silicate lattice under strong alkaline conditions. For the ZMM9 sample (9 M), the Zn–O and Mn–O bands are clearly defined, while the montmorillonite O–H band is significantly suppressed, which may be a consequence of the basicity of the solution. Additionally, the slight change in the metal–oxygen region can be linked to interfacial Zn–O–Mn linkages or highly hydroxylated metal oxides that form under these conditions.^37^ Overall, the progressive changes in the FTIR spectra with increasing NaOH suggest that a higher base accelerates precursor hydrolysis and nucleation of ZnO/MnO_2_, promotes cation exchange into the clay mineral interlayers, and drives the reorganization of both the clay mineral and oxide surface hydroxyl lattices. These coupled effects coherently explain the observed FTIR evolution.

### 3.2 Optical Analyses of the ZnO@MnO_2_-Montmorillonite Nanocomposite

The effect of precursor molarity on defect formation in the ZnO@MnO_2_-montmorillonite nanocomposite was also investigated by photoluminescence spectroscopy. Figure S2 (Supporting Information) displays the combined photoluminescence spectra of the ZMM3, ZMM5, ZMM7, and ZMM9 nanocomposites. In contrast, Figure 2a–d presents the room-temperature photoluminescence spectra of each sample separately, enabling a clearer comparison of their emission characteristics. In ZnO-based semiconductors, emissions appear in two distinct regions: a sharp ultraviolet band originating from excitonic recombination at the band edge (NBE), and a broader visible band arising from defect-related transitions.^32,42,43^ Analyzing the peak in the UV region of the PL spectra, we see a change in shape and position, which may be related to the effects of precursor concentration on nucleation and growth and the microstructure of the nanocomposite. In this sense, a higher solution molarity increases supersaturation during synthesis, promoting rapid nucleation and resulting in smaller ZnO crystallites. The quantum-confinement effect increases the recombination energy by spatially restricting electrons and holes to reduced volumes, thereby shifting the excitonic peak. At the same time, elevated molarity enhances the density of surface imperfections and heterointerfaces with montmorillonite, which perturbs the local electric field and lattice symmetry; these additional defect-induced energy levels near the conduction or valence band further fine-tune the ultraviolet peak energy.

**Figure 2:**
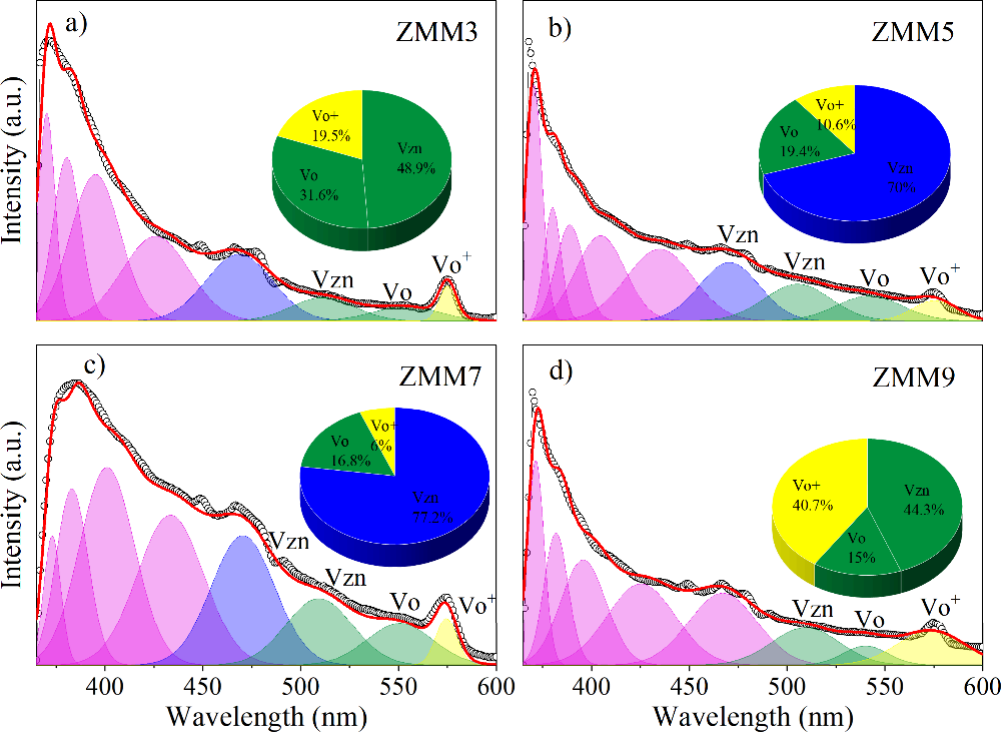
Photoluminescence spectra for the ZnO@MnO_2_-montmorillonite nanocomposite. (a) ZMM3, (b) ZMM5, (c) ZMM7 and (d) ZMM9. The inset shows the defect concentration quantified by the area under the curve, which was determined by fitting a Gaussian function.

Similar results are also observed in other studies where the pH conditions of the synthesis process were changed.^12,32,44^ Conversely, the photoluminescence spectra were deconvoluted using a Gaussian function, with emphasis on the broad band in the visible region associated with intrinsic defect centers. This fitting procedure quantifies, via the integrated area under each Gaussian peak, how variations in precursor molarity modulate the composite’s population of structural defects.

The pie charts in Figure 2a–d illustrate the primary defects quantified in the visible region of the analyzed spectrum. As observed, the ZMM3 sample (3 M) exhibits a distribution of ∼49% zinc vacancies (*V*_Zn_), ∼32% neutral oxygen vacancies (*V*_O_), and ∼20% ionized oxygen vacancies (*V*_O_^+^). Increasing the molarity to 5 M (ZMM5) and 7 M (ZMM7) causes a significant increase in the proportion of *V*_Zn_, reaching approximately 70% and 77%, respectively. This is accompanied by a notable decrease in both neutral and ionized oxygen vacancies, which together account for less than 20%. This trend suggests that strongly alkaline conditions promote rapid precipitation of Zn­(OH)_2_, resulting in ZnO enriched with zinc-related defects, likely due to enhanced Zn^2^^+^ incorporation into the interlayer cationic sites of montmorillonite and structural rearrangements at the ZnO/MnO_2_/clay mineral interfaces. As a result, the ZMM5 sample and particularly the ZMM7 sample exhibit intensified ultraviolet emission near the band-to-band transition and prominent green emission bands associated with *V*_Zn_, consistent with literature reports attributing ZnO’s intrinsic green luminescence to zinc vacancies.^45^ In contrast, the ZMM9 sample (9 M) exhibits a sharp increase in the proportion of ionized oxygen vacancies (*V*_O_^+^, ∼40.7%) and a corresponding decrease in zinc vacancies (*V*_Zn_, ∼44%), indicating that the highly alkaline environment promotes partial dissolution of ZnO and leads to unoccupied oxygen sites. This behavior is evident in the photoluminescence spectrum of the ZMM9 sample, which is dominated by a green–yellow emission band characteristic of oxygen-related defects. This observation aligns with previous reports that attribute yellow-to-orange emissions to the presence of oxygen vacancies or excess oxygen species.^45^ Furthermore, the overall emission intensity is lower, suggesting increased nonradiative recombination routes. For practical properties, a material enriched with zinc vacancies typically exhibits enhanced optoelectronic performance, such as increased emission efficiency and potential p-type conductivity, due to the altered density of states within the band gap. Conversely, the synergistic effect between cationic (*V*_Zn_) and anionic defects (*V*_O_) generally promotes improved photocatalytic activity and more effective charge separation.^46^ Additionally, a higher concentration of oxygen vacancies can enhance electronic conductivity and facilitate redox reactions in electrochemical applications, such as photocatalysis and energy storage electrodes, owing to the increased availability of reactive oxygen sites. Therefore, tuning the NaOH molarity establishes a direct correlation between the composite’s microstructure, characterized by techniques such as X-ray diffraction and Fourier transform infrared spectroscopy, and its electronic defect profile, enabling the complementary optimization of its optoelectronic, photocatalytic, and electrochemical properties.

On the other hand, the band gap energy of the ZnO@MnO_2_-Montmorillonite nanocomposite was estimated by combining the Kubelka–Munk function with the Tauc plot,^42,47–50^ based on the diffuse reflectance spectra presented in Figure 3a. The calculated band gap values in Figure 3b reveal a systematic decrease with increasing NaOH molarity, from 3.294 eV for the ZMM3 sample to 2.945 eV for the ZMM9 sample. This shift toward the visible region may be attributed to chemical and physicochemical changes induced by the increasing alkalinity of the reaction medium, which enhances the relative concentrations of structural defects, namely zinc vacancies and both neutral and ionized oxygen vacancies, as evidenced by the PL deconvolution spectra. These defects introduce intermediate electronic states within the band gap, broadening the absorption tail and effectively narrowing the band gap, a phenomenon similar to that observed in ZnO systems enriched with zinc vacancies.^45,51,52^

**Figure 3:**
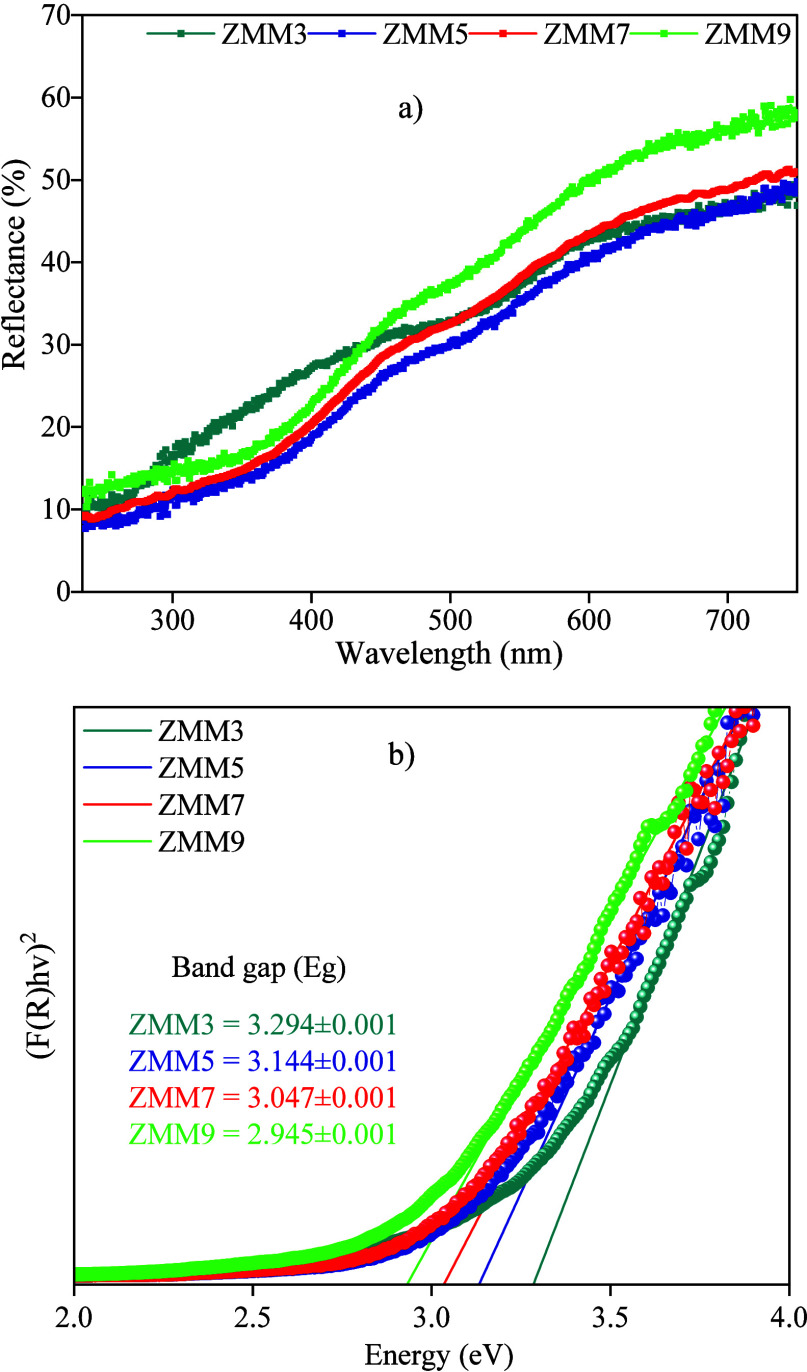
(a) DRS spectra and (b) calculated band gap values for the ZnO@MnO_2_-Montmorillonite nanocomposite.

Furthermore, the triphasic composition of the material enhances this optical variation: while ZnO serves as the primary semiconductor matrix, the dispersed MnO_2_ phase increases surface and interfacial disorder, due to crystallographic and electronic mismatches with ZnO, thereby generating additional defect centers. Meanwhile, the montmorillonite host, characterized by its high cation exchange capacity, adsorbs Zn^2^^+^ and Mn^2^^+^ ions, leading to ionic vacancies and lattice distortions within the ZnO structure. As a result, the strong structural and electronic interactions among ZnO, MnO_2_, and the clay mineral induce crystalline strain and heterointerfacial band misalignment, which sharpen the absorption edge (Urbach tail) and enable fine modulation of the nanocomposite’s energy gap through defect engineering and interfacial transitions.

### 3.3 Morphological and Porosity Studies of the ZnO@MnO_2_-Montmorillonite Nanocomposite

The morphology of the ZnO@MnO_2_-montmorillonite nanocomposites was examined by scanning electron microscopy (SEM), and the microstructural evolution as a function of solution molarity is shown in Figure 4a–d. Across all samples, the clay provides a lamellar scaffold on which oxide crystallites nucleate and grow. As noted, the ZMM3 sample (3 M) exhibits a homogeneous microstructure (Figure 4a), in which large montmorillonite platelets remain structurally intact and well-defined, serving as stable lamellar supports. These surfaces are uniformly decorated with submicrometric aggregates, likely ZnO and MnO_2_ nanocrystals, anchored through heterogeneous nucleation. Such a configuration is indicative of a moderate nucleation rate under this molarity, which ensures sufficient supersaturation to initiate oxide precipitation while allowing controlled growth and uniform dispersion across the accessible sites of the layered matrix. This balance preserves platelet morphology and maximizes interfacial contact between the clay substrate and the oxide phases, potentially enhancing the hybrid’s functional properties.

**Figure 4:**
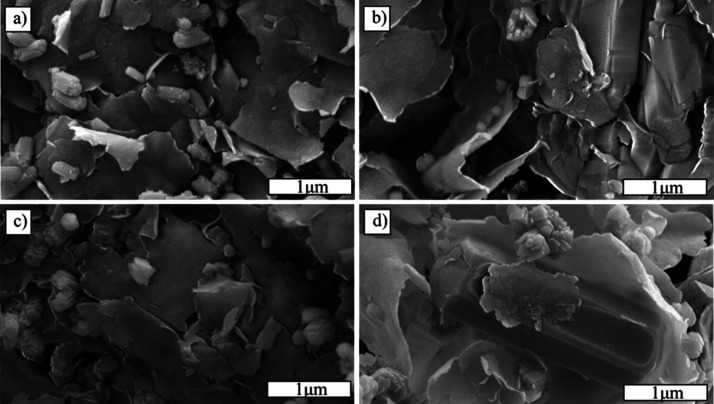
SEM micrographs of ZnO@MnO_2_-montmorillonite nanocomposites synthesized at different molarities: (a) ZMM3 (3 M), homogeneous distribution of nanocrystals over well-defined clay platelets; (b) ZMM5 (5 M), denser coverage and localized coalescence of oxide domains; (c) ZMM7 (7 M), thicker, continuous oxide coatings with partial masking of the clay surface; (d) ZMM9 (9 M), anisotropic rod-like crystallites and heterogeneous aggregates adhering to the lamellar substrate.

In contrast, the ZMM5 sample (5 M) exhibits more extensive surface coverage and localized coalescence of the oxide phase (Figure 4b). Montmorillonite platelets become partially masked by denser clusters, and particle necking is evident along platelet edges and terraces. These features suggest the onset of sintering or oriented coalescence, driven by higher supersaturation that accelerates Zn­(OH)_2_ precipitation and its rapid conversion to ZnO. This kinetic regime promotes the formation of well-connected oxide domains with intimate contact to the clay substrate, potentially enhancing interfacial charge transfer and light-harvesting efficiency, factors that are consistent with the superior photocatalytic performance observed for this composition. On the other hand, the ZMM7 sample (7 M) displays a more compact deposit structure (Figure 4c). The oxide phase develops into thicker, more continuous coatings over the clay platelets, indicating a predominance of lateral growth and coalescence over fresh nucleation. This results in diminished exposure to the underlying layered surface and suggests partial masking or encapsulation of the montmorillonite, accompanied by increased interparticle sintering. Such morphological consolidation can limit the accessibility of certain adsorption sites and alter mass-transfer dynamics, potentially hindering photocatalytic efficiency compared with the more open and well-dispersed architecture of ZMM5.

Finally, the ZMM9 sample (9 M) exhibits a marked transition toward anisotropic architecture (Figure 4d). Elongated, rod-like crystallites are interspersed with rough, irregular aggregates intimately attached to the clay platelets. Such anisotropic growth is characteristic of rapid crystallization kinetics under highly alkaline conditions, where elevated [OH^–^] facilitates oriented attachment and facet-selective growth, favoring elongation along specific crystallographic directions. The presence of heterogeneous aggregates suggests concurrent secondary nucleation events and possible reprecipitation processes, which can generate structural disorder at the oxide–clay interface. While SEM alone cannot conclusively determine phase identity, the rod-like habit is compatible with accelerated ZnO growth, and the intimate adherence to the lamellar substrate indicates strong interfacial coupling within the hybrid framework.

On the other hand, the influence of solution molarity on the porosity of the ZnO@MnO_2_-Montmorillonite nanocomposite was investigated from the isotherms of N_2_ adsorption/desorption (Figure 5a). All samples present nitrogen adsorption/desorption isotherms that can be classified as type IV, with hysteresis of type H4,^53^ characteristics of mesoporous solids with layered structure and slit-shaped pores, characteristics intrinsic to montmorillonite and reinforced by the presence of metal oxides.

**Figure 5:**
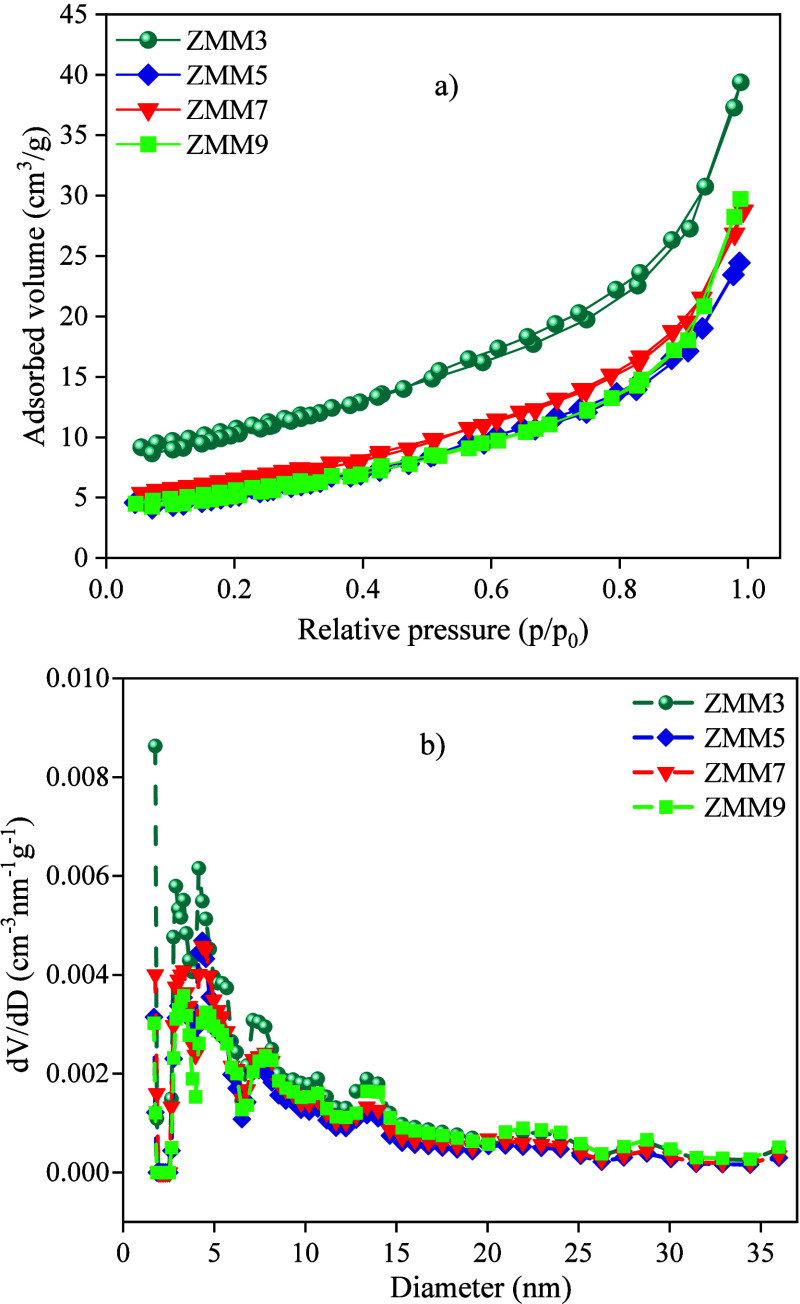
(a) N_2_ adsorption/desorption and (b) pore distribution for the ZnO@MnO_2_-montmorillonite nanocomposite.

Furthermore, all samples show a similar pore distribution with a pronounced peak between ∼1.7 and ∼4.5 nm, depending on the molarity of the solution (Figure 5b). Table 2 presents the textural parameters for the ZnO@MnO_2_-Montmorillonite nanocomposite. As noted, the ZMM3 sample exhibits the highest BET surface area (∼35 m^2^.g^–1^) and the smallest average pore diameter (∼1.76 nm), indicating a predominantly microporous structure. In contrast, the ZMM5, ZMM7, and ZMM9 samples show significantly lower surface areas (∼18–22 m^2^.g^–1^) and larger average pore diameters (∼3.3–4.3 nm), as evidenced by the reduced intensity of the N_2_ adsorption isotherms and the shift in pore size distribution toward wider ranges.

**Table 2:** Textural Parameters for the ZnO@MnO_2_-Montmorillonite Nanocomposites

samples	**area** **(m** ^**2**^ **g** ^**–**^ **^1^)** **B.E.T. method**	**pore volume** **(cm** ^**3**^ **g** ^**–**^ **^1^)** **NLDFT method**	average pore diameter (nm) NLDFT method
ZMM3	35.1	0.052	1.76
ZMM5	18.8	0.037	4.32
ZMM7	21.8	0.32	4.34
ZMM9	18.7	0.038	3.32

This phenomenon can be attributed, first, to the strong alkaline environment above the montmorillonite: under very high pH, preferential dissolution of the aluminum-silicate edges can occur, reducing the volume of the grains and disrupting the lamellar stacking, so that the clay mineral particles become monolayers without three-dimensional periodicity.^54^ Furthermore, the ZnO formation and the MnO_2_ incorporation under highly alkaline conditions can lead to the creation of irregular aggregates, which block the original micropores or act as disordered pillars. It is well established, for instance, that increasing the metal oxide content in montmorillonite-based heterostructure materials results in a simultaneous decrease in BET surface area and microporous fraction, an effect attributed to pore blockage by irregularly shaped porous structures.^9^ In addition, the uncontrolled precipitation of these oxides can result in an architecture dominated by nonuniform mesopores, which explains the low specific surface area observed in composites synthesized with high NaOH concentrations.^9^ In summary, increasing the NaOH molarity likely promotes the partial dissolution of the montmorillonite matrix in the ZnO@MnO_2_-Montmorillonite nanocomposite, along with a concomitant reorganization of the pore structure, from a dense microporous lattice (3 M) to a more open mesoporous architecture (5–9 M). This results in a reduction in surface area and an increase in average pore diameter, as confirmed experimentally. These results are consistent with reports in the literature on clay mineral-based materials activated under strongly alkaline conditions and in the presence of metal oxides.^9,54^

### 3.4 Photocatalytic Tests

The photocatalytic degradation of ciprofloxacin (CIP) was performed in a borosilicate reactor connected to a thermostatic bath, which was maintained at a temperature of 24 °C ± 1 °C. A 125 W UV lamp (emission peak at 350–450 nm, and light intensity of 6.0 ± 0.2 μW cm^2^) was utilized as the irradiation source in the experimental setup. The reaction system comprised 0.5 g L^–1^ of photocatalyst and 20 mg L^–1^ of the CIP pollutant. Initially, the system was kept in the dark for 30 min to establish adsorption/desorption equilibrium. After this period, the UV lamp was activated. Aliquots were collected at designated time intervals (0, 10, 20, 30, 45, 60, 90, and 120 min) for analysis. The absorbance of the CIP drug was measured at 275 nm using a Shimadzu UV-2700 spectrophotometer. The degradation efficiency was calculated using the following equation (eq 1):

$$\mathrm{Degradation}\;(\%)=(C_{0}-C)/C_{0}\times 100$$ 1

Where *C*_0_ denotes the initial concentration and C represents the final concentration of the contaminant.

Photocatalytic experiments were carried out to evaluate the performance of ZnO@MnO_2_-montmorillonite nanocomposites in degrading ciprofloxacin (CIP) under UV irradiation for 120 min. Figure 6 shows the time-dependent CIP removal profiles and the corresponding degradation efficiencies: 60.1% (ZMM3), 61.0% (ZMM5), 59.7% (ZMM7), and 59.5% (ZMM9).

**Figure 6:**
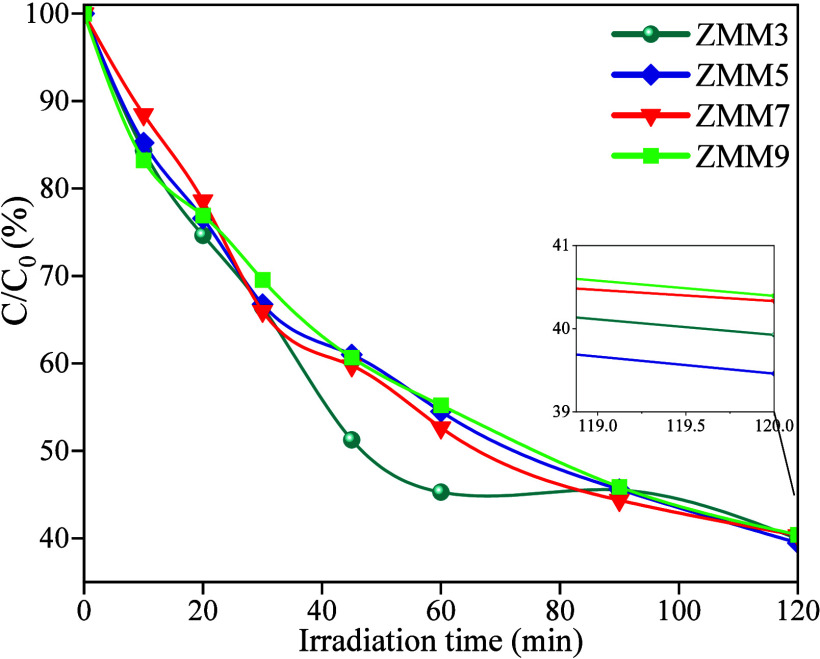
*C*/*C*_0_ versus irradiation time for ciprofloxacin removal using ZnO@MnO_2_-montmorillonite nanocomposites (ZMM3, ZMM5, ZMM7, and ZMM9). The inset shows a magnified view of the final stage of the degradation (between 119 and 120 min), highlighting the residual concentration differences among the samples.

Although the differences among samples are relatively small, all compositions exhibit significant photocatalytic activity, indicating that the hybrid structure, comprising ZnO and MnO_2_ nanoparticles anchored on the montmorillonite surface, effectively promotes CIP degradation under UV light. The comparable efficiencies suggest that, within the tested molarity range, variations in precursor concentration do not markedly affect UV-driven performance, likely due to similar band gap values and strong oxide–clay interfacial coupling across the series. This interpretation is further corroborated by the pseudo-first-order kinetic analysis.

As shown in Figure 7, the linear relationship between ln­(*C*/*C*_0_) and irradiation time confirms that the apparent rate constants (*k*), calculated using the Langmuir–Hinshelwood (L–H) model, are comparable for all nanocomposites. This similar kinetic behavior suggests that their photocatalytic activity arises from shared structural features, surface characteristics, and optical properties, which collectively govern light absorption, charge separation, and reactant interaction at the catalyst interface. The hydrothermal synthesis of the nanocomposite yields a highly photoactive and stable material enriched with structural defects, including both anionic and cationic vacancies, as evidenced by PL analysis. These defects, particularly when located at or near the surface, act as active sites that enhance light harvesting and facilitate the adsorption and activation of reactant molecules. Moreover, they serve as charge-trapping centers that inhibit rapid electron–hole recombination, thereby promoting efficient charge separation and accelerating the photocatalytic degradation process.^55,56^

**Figure 7:**
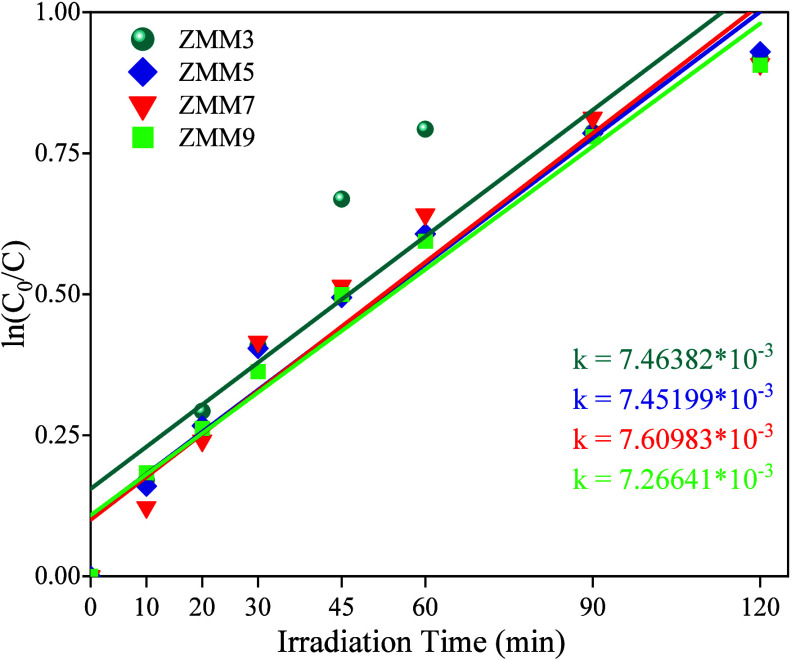
Kinetic profiles for ciprofloxacin removal under UV irradiation using ZnO@MnO_2_-montmorillonite nanocomposites (ZMM3, ZMM5, ZMM7, and ZMM9).

Moreover, structural defects can introduce intermediate energy states within the band gap, facilitating sub-bandgap excitation and suppressing rapid electron–hole recombination, thus enhancing photocatalytic efficiency.^57–59^ The montmorillonite component, with its high surface reactivity, not only supports uniform nanoparticle dispersion but also improves sorption–diffusion processes and promotes efficient charge transfer at the catalyst–pollutant interface.^60^ Such oxide–clay heterostructures display synergistic effects, where the intimate coupling between metal oxide nanoparticles and the layered clay matrix increases the density of active sites, optimizes carrier dynamics, and enhances both the stability and functionality of the photocatalyst.^34,35,57,60–62^

Additional tests were conducted to evaluate the operational parameters involved in the photodegradation of CIP (Figure 8). The efficiency of the process is influenced by key parameters, such as photocatalyst dosage, initial contaminant concentration, and the pH of the medium.^21,26,34,63,64^ This study aims to assess the influence of these variables on the degradation of ciprofloxacin and optimize conditions for applications in wastewater treatment. In this sense, tests were performed by varying the following parameters: photocatalyst concentration (25, 50, and 100 mg L^–1^) to determine the saturation of active sites; CIP concentration (10, 20, and 30 mg L^–1^) to evaluate competition for oxidizing radicals; and pH (3, 5, and 9) to investigate its effects on the surface charge of the catalyst and CIP ionization.

**Figure 8:**
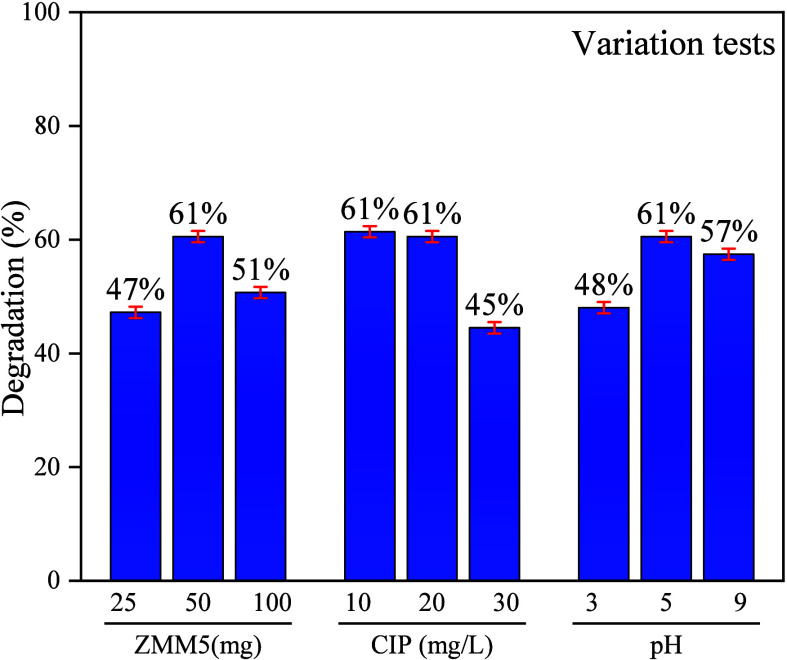
Influence of operational parameters on ciprofloxacin degradation efficiency using the ZMM5 nanocomposite. The graph presents the effects of catalyst dosage (25, 50, and 100 mg), initial ciprofloxacin concentration (10, 20, and 30 mg L^–1^), and solution pH (3, 5, and 9) on the residual concentration ratio (*C*/*C*_0_ %), after 120 min of irradiation. Error bars represent the standard deviation from triplicate experiments.

The results indicated that changes in catalyst loading led to a slight reduction in activity at both 25 mg L^–1^ and 100 mg L^–1^ concentrations, while 50 mg L^–1^ proved to be the ideal concentration for removing 61% of the drug from the solution. This suggests that a lower catalyst concentration limits the availability of active sites, and a higher concentration might affect the light absorption properties of the semiconductor due to nanoparticle agglomeration in the solution. From varying the CIP concentrations, we concluded that a concentration of up to 20 mg L^–1^ enhances the removal of molecules (61%).

However, higher concentrations partially decrease the photocatalytic potential of the material (45%). This reduction is likely due to increased adsorption of molecules on the catalyst surface, a higher concentration of byproducts, and reduced photon absorption capacity, resulting in fewer reactive species. The pH variation also had a slight effect on the material’s activity. At pH 3, electrostatic repulsion may occur between the positively ionized CIP molecules and the surface of the nanocomposite, slightly reducing the adsorption and photodegradation of drug molecules up to 48%. At pH 5 (61% of the degradation), CIP exists in its zwitterionic form, which favors interactions with the surface of the nanocomposite, enriched with Zn^2^^+^ and Mn^2^^+^ ions. Following this trend, at pH 9, where CIP is negatively charged, electrostatic forces can influence interfacial interactions and promote the oxidative degradation process of the semiconductor, achieving 57% removal. Overall, the results indicated that even under optimized conditions, variations in these operational parameters did not significantly affect the removal of the drug using the ZMM5 catalyst. This suggests that the material is promising for practical applications, as it has demonstrated efficiency across various scenarios. Therefore, it is a versatile, stable, economical, and scalable alternative for use in real systems with complex matrices that can alter ideal conditions.

Inhibitor tests were conducted to clarify the photodegradation mechanism and identify the reactive species involved, including hydroxyl radicals (•OH), superoxide anions (•O_2_^–^), holes (h^+^), and electrons (e^–^), as shown in Figure 9. Specific scavengers were introduced into the initial reaction mixture to quench the targeted reactive species: - To target electrons, 0.0086 g of silver nitrate (AgNO_3_) was added. To target holes, 0.0075 g of ethylenediaminetetraacetic acid (EDTA) was incorporated.

**Figure 9:**
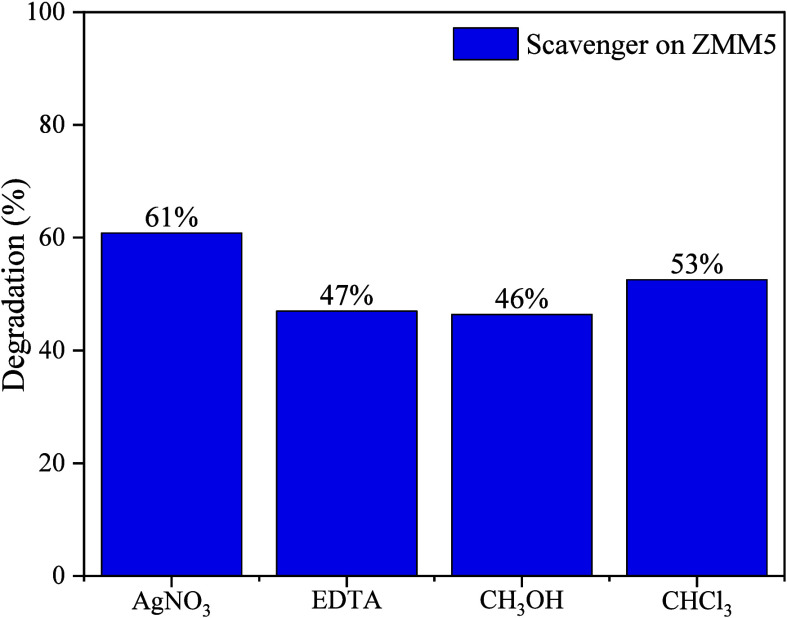
Photocatalytic scavenger tests for ciprofloxacin degradation using the ZMM5 nanocomposite. Various quenchers, AgNO_3_ (electron scavenger), EDTA (hole scavenger), CH_3_OH (hydroxyl radical scavenger), and CHCl_3_ (superoxide radical scavenger), were employed to elucidate the active species involved in the reaction.

To target hydroxyl radicals, 406 μL (CH_3_OH) was introduced. To target superoxide radicals, 406 μL of chloroform (CHCl_3_) was added. After the addition of each respective scavenger, the suspension was exposed to UV light irradiation. The procedure followed was similar to that used in the photocatalytic tests. These tests are crucial for understanding the degradation of molecules and for optimizing catalyst efficiency. By evaluating the role of each active species, we can confirm the primary degradation pathway of the target pollutant. The results reveal that the addition of silver nitrate to the system does not lead to any significant change in photocatalytic activity, suggesting that electrons may not play a direct role in the photocatalysis process. However, when EDTA, methanol, and chloroform are introduced, the degradation rates decrease to 47%, 46%, and 53%, respectively. These findings highlight the involvement of species such as h^+^, •OH, and •O_2_^–^ (mainly •OH and h^+^) in the degradation mechanism of ciprofloxacin using the ZMM5 material. This is consistent with existing literature,^34,65^ emphasizing that hydroxyl radicals and holes, as oxidizing agents, are the primary species driving the degradation of the CIP drug. These reactive species courageously target the piperazine moiety, leading to the transformative destruction and cleavage of the piperazine rings.^22,26^ It is known that photochemical reactions occur at the interface between a solid semiconductor surface and a liquid surrounding medium, which includes reagents and reaction products/byproducts.^66^ The overall process can be outlined as follows: i. Excitation of the ZMM5 semiconductor by light. ii. Generation of electron–hole pairs (e^–^/h^+^). iii. Migration of these charge carriers to the surface of the semiconductor, where they participate in redox reactions. iv. Formation of reactive oxygen species. v. Decomposition of drugs present in the solution into less toxic byproducts, such as CO_2_ and H_2_O. Thus, the degradation mechanism of ciprofloxacin using ZMM5 catalyst can be effectively summarized by the following eqs 2–8 and represented in Figure 10.

$$\mathrm{ZMM}5+hv\to {e^{-}}_{\left(\mathrm{CB}\right)}+{h^{+}}_{\left(\mathrm{VB}\right)}$$ 2

$${h^{+}}_{\left(\mathrm{VB}\right)}+H_{2}O\to •\mathrm{OH}+H^{+}$$ 3

$${h^{+}}_{\left(\mathrm{VB}\right)}+{\mathrm{OH}}^{-}\to •\mathrm{OH}$$ 4

$${e^{-}}_{\left(\mathrm{CB}\right)}+O_{2}\to •{O_{2}}^{-}$$ 5

$$•\mathrm{OH}+\mathrm{CIP}\to \mathrm{harmless}\;\mathrm{products}+{\mathrm{CO}}_{2}+H_{2}O$$ 6

$$h^{+}+\mathrm{CIP}\to \mathrm{harmless}\;\mathrm{products}+{\mathrm{CO}}_{2}+H_{2}O$$ 7

$$•{O_{2}}^{-}+\mathrm{CIP}\to \mathrm{harmless}\;\mathrm{products}+{\mathrm{CO}}_{2}+H_{2}O$$ 8

**Figure 10:**
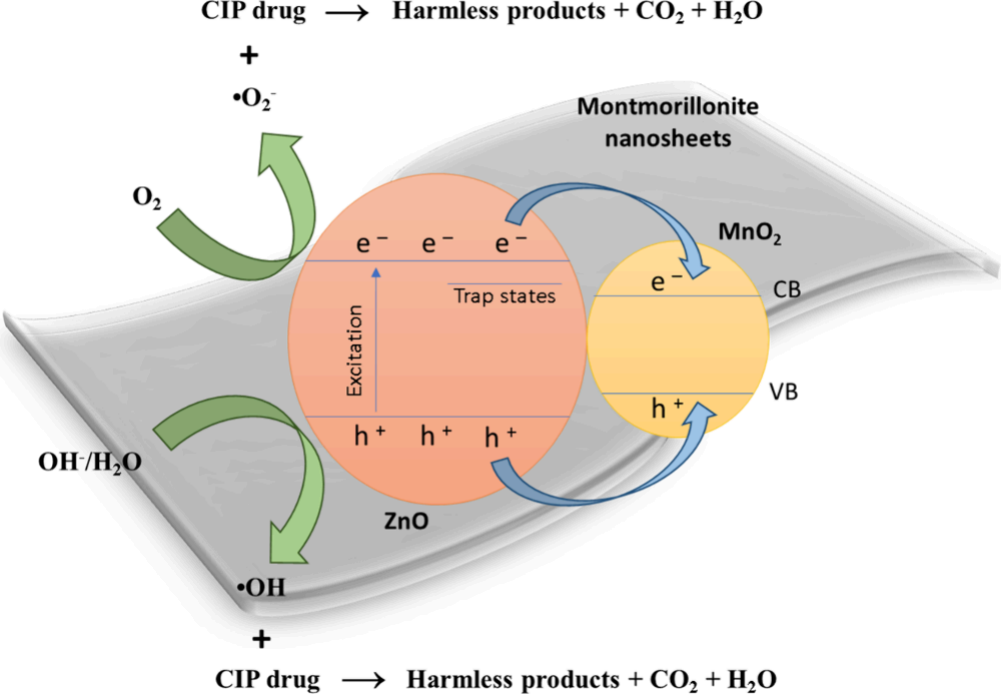
Schematic representation of the proposed photocatalytic mechanism for ciprofloxacin degradation using ZnO@MnO_2_-montmorillonite nanocomposites.

The reuse of semiconductors has emerged as a significant area of research, particularly because of their practical applications in sensors, optoelectronic devices, solar cells, and photocatalysis.^67–70^ In photocatalysis testing, the emphasis is on assessing the stability, efficiency, and performance of the material after undergoing multiple reuse cycles. The tests were conducted under identical conditions to the previous photocatalytic tests. After each cycle, the ZMM5 nanocomposite was recovered by centrifugation, dried, and reused without any chemical regeneration. For the ZMM5 sample, three consecutive reuse cycles were conducted, as shown in Figure 11. The results indicate that the material exhibits good reusability after these three cycles, although its efficiency decreases by approximately 50%.

**Figure 11:**
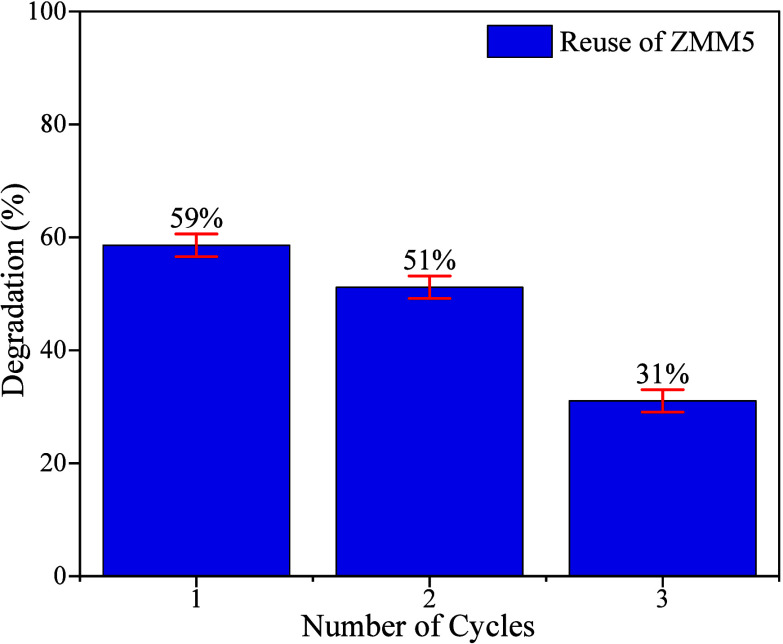
Reusability performance of the ZMM5 nanocomposite for ciprofloxacin degradation under UV irradiation across three consecutive cycles. Error bars represent standard deviations from triplicate measurements.

XRD measurements were performed to evaluate the stability of the nanocomposite following reuse. Figure 12 presents the diffractogram of sample ZMM5 both before and after the reuse photocatalytic tests. All peaks corresponding to the ZnO phases ((100), (002), (101), (102), (110), (103), (112), and (201)), MnO_2_ phases ((211), (301), (521), (541), and (312)), and montmorillonite phases ((110), (004), (105), (201), and (060)) were observed after three consecutive testing cycles, in comparison to the sample analyzed before the photocatalytic tests. Importantly, no new peaks appeared, indicating that there was no formation of secondary phases or alterations in the crystalline structure. These results underscore the stability of the material, demonstrating that the crystalline structure remains intact after reuse. Consequently, the nanocomposite exhibits significant stability and reusability, positioning it as a promising candidate for practical applications.

**Figure 12:**
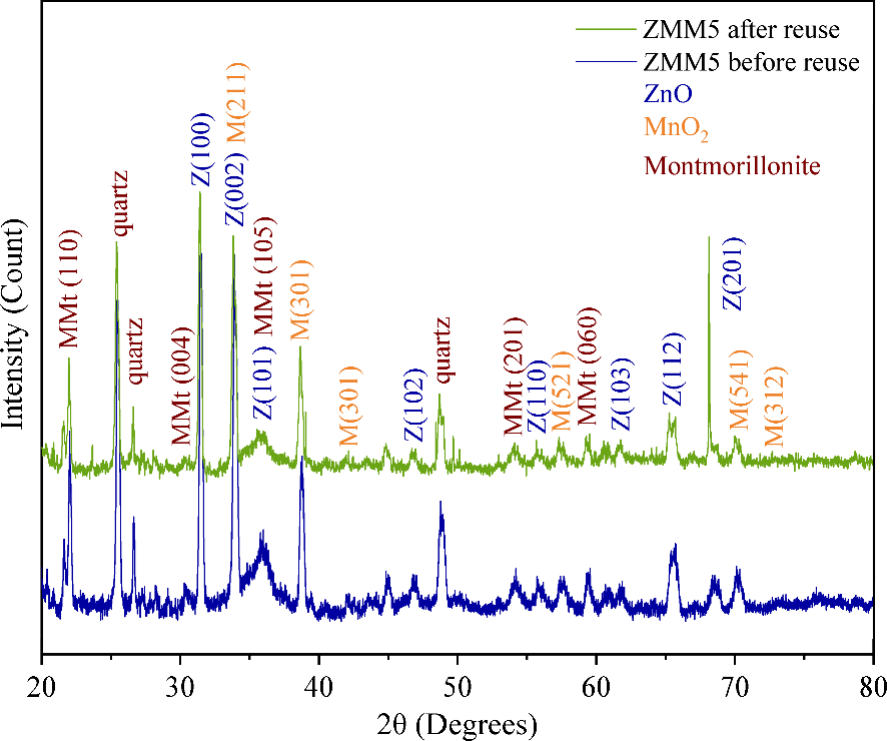
XRD patterns of the ZMM5 nanocomposite before and after reuse in the photocatalytic degradation of ciprofloxacin under UV light.

## 4 Conclusions

This work presents a comprehensive approach to defect and interface engineering in ZnO@MnO_2_-Montmorillonite nanocomposites synthesized under controlled alkaline conditions. By varying the NaOH molarity from 3 to 9 M, it was possible to tailor the crystallite size, dislocation density, surface area, and defect profile, parameters that directly influence the optical response and photocatalytic behavior of the material. Despite differences in microstructure, all samples exhibited consistent photocatalytic degradation of ciprofloxacin, highlighting their robustness and versatility. The ZMM5 sample, synthesized at 5 M, emerged as the optimal formulation, balancing crystallinity, defect density, and surface characteristics to achieve 61% degradation in 120 min under UV light. Additionally, it maintained high performance across a range of operating conditions (pH, pollutant concentration, and catalyst dosage), demonstrating its potential for real-world wastewater applications. The structural stability confirmed by XRD after three reuse cycles reinforces the material’s reusability and operational resilience. Altogether, the study underscores the importance of molarity control in hydrothermal synthesis as a strategic parameter for tuning photocatalytic nanocomposites and highlights ZnO@MnO_2_-Montmorillonite systems as scalable and environmentally promising materials for the treatment of pharmaceutical contaminants.

## Supplementary Material



## Data Availability

All the data required to reproduce these experiments are present in the article.

## Notes

X-ray diffraction patterns of the starting materials (ZnO, MnO_2_, and montmorillonite), highlighting their crystalline phases, as well as comparative photoluminescence spectra of the ZnO@MnO_2_-montmorillonite nanocomposites (without Gaussian deconvolution), providing complementary structural and optical characterization that support the discussion in the main manuscript (PDF)
