# Supplementary material for: Hydrothermal Synthesis of ZnO@MnO2‑Montmorillonite Nanocomposites: Influence of Molarity on Structural, Optical, and Photocatalytic Performance toward Ciprofloxacin Degradation under Variable Conditions
Source: ACS Omega. 2025 Sep 19;10(38):44461–74. doi: 10.1021/acsomega.5c06454 (PMC12489627; doi:10.1021/acsomega.5c06454)
Supplement: Supplementary file 1 [file ao5c06454_si_001.pdf]

Supplementary Material

for

# Hydrothermal synthesis of $ZnO@MnO_2$-Montmorillonite nanocomposites: Influence of molarity on structural, optical, and photocatalytic performance toward Ciprofloxacin degradation under variable conditions

Elisabethe Bezerra[1], Willams A. Albuquerque[2], Adilson J. Neres Filho[1], Alexsandro Lins[2], Ricardo Barbosa[1], Luciano Almeida[3], Santiago Medina-Carrasco[4], Maria del Mar Orta Cuevas[5], Josy A. Osajima[6], Pollyana Trigueiro[1], Ramón Raudel Peña Garcia[1,2,6 *]

[1]*Universidade Federal Rural de Pernambuco, Programa de Pós-Graduação em Engenharia Física, Unidade Acadêmica do Cabo de Santo Agostinho, 54518-430, Cabo de Santo Agostinho-PE, Brazil*

[2]*Universidade Federal de Pernambuco, Programa de Pós-Graduação em Ciência de Materiais, 50740-560, Recife-PE, Brazil.*

[3]*Universidade Federal de Pernambuco, Departamento de Engenharia Química, 50.740-590, Recife-PE, Brazil*

[4]*SGI Laboratorio de Rayos X - Centro de Investigación, Tecnología e Innovación de la Universidad de Sevilla (CITIUS), 41012, Sevilla, España*

[5]*Universidad de Sevilla, Departamento de Química Analítica, Facultad de Farmacia, 41012, Sevilla, España*

[6]*Laboratório Interdisciplinar de Materiais Avançados (LIMAV), Centro de Tecnologia, 64049-550, Teresina- PI, Brazil*

***Corresponding authors**: Ramón Raudel Peña Garcia (rraudelp@gmail.com; ramon.raudel@ufrpe.br).

**Figure S1** presents the X-ray diffraction patterns of the starting materials utilized in synthesizing the nanocomposite. The analysis demonstrates the presence of three distinct crystalline phases: ZnO (hexagonal, JCPDS 36-1451), $MnO_2$ (tetragonal, JCPDS 44-0141), and montmorillonite (JCPDS 03-0015). ZnO displays characteristic peaks at $2\theta$ = 31.84$^0$, 34.58$^0$, 36.31$^0$, 47.61$^0$, 56.68$^0$, 62.95$^0$, 66.42$^0$, 67.98$^0$, 69.09$^0$, 72.55$^0$, and 76.92$^0$, corresponding to the crystallographic planes (100), (002), (101), (102), (110), (103), (200), (112), (201), (004), and (202) [1]. $MnO_2$ shows peaks at $2\theta$ = 34.64$^0$, 40.56$^0$,

$58.78^{0}$, $70.26^{0}$, and $73.79^{0}$, matching the (211), (301), (521), (541), and (312) planes [2]. Montmorillonite exhibits crystalline peaks at $2\theta = 20.17^{0}$, $29.12^{0}$, $35.38^{0}$, $54.98^{0}$, and $62.22^{0}$, corresponding to the (110), (004), (201), and (060) reflection planes [3]. Additionally, natural montmorillonite presents characteristic quartz (Q) peaks at $2\theta$ = $27.16^{0}$ and $28.52^{0}$.

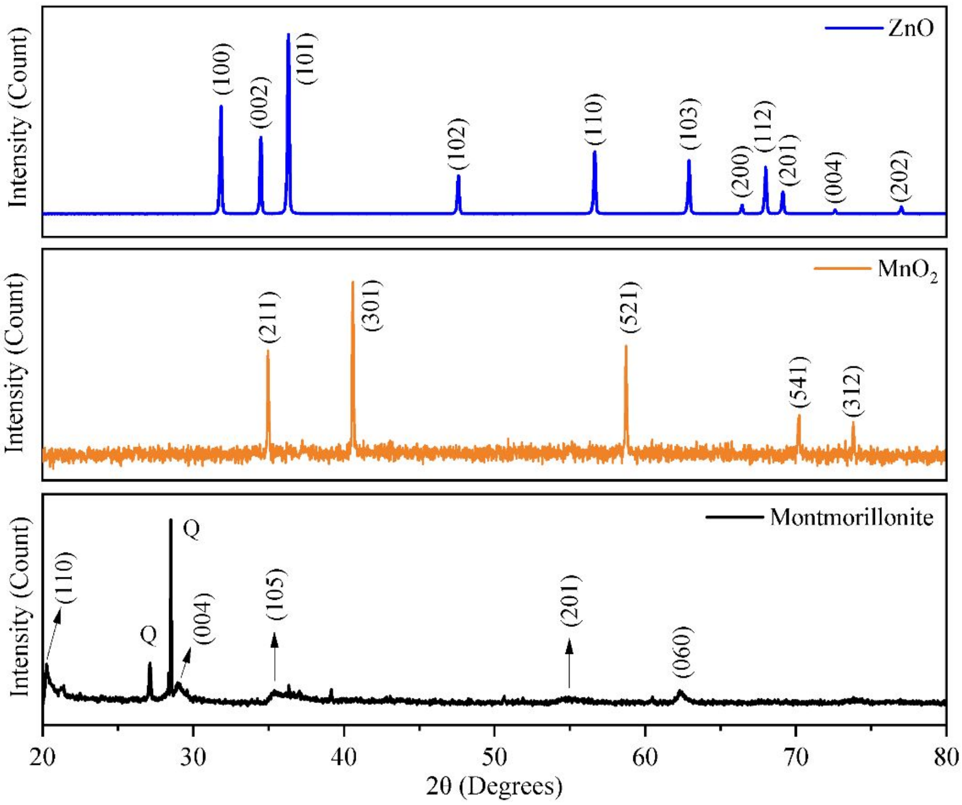


**Figure S1.** XRD patterns of bare ZnO, $MnO_2$ and montmorillonite structures.

**Figure S2** presents the UV emission spectra for nanostructures.

UV Emission Region (350–450 nm): The prominent peak is attributed to near-band-edge (NBE) emission, arising from excitonic recombination just below the band edge for all samples. ZMM5 and ZMM9 exhibited strong NBE emission and relatively weak visible luminescence, indicating high crystallinity and a low concentration of intrinsic defects. However, in ZMM3 and ZMM9, significant modifications in the UV emission

characteristics were observed—the NBE emission was quenched many times. This reduction is attributed to the formation of non-radiative recombination centers [1,4,5].

Visible Emission Region (450–600 nm): This broad emission range corresponds to several intrinsic point defect states within the ZnO-based nanocomposites band gap. The visible emission spectra were deconvoluted using Gaussian fitting to analyze these defect states, with particular focus on the broad band associated with intrinsic defect centers. The integrated area under each Gaussian peak quantifies how variations in precursor molarity influence the nanocomposite's structural defect population. The main manuscript provides a comprehensive explanation of the defect dynamics [6–9].

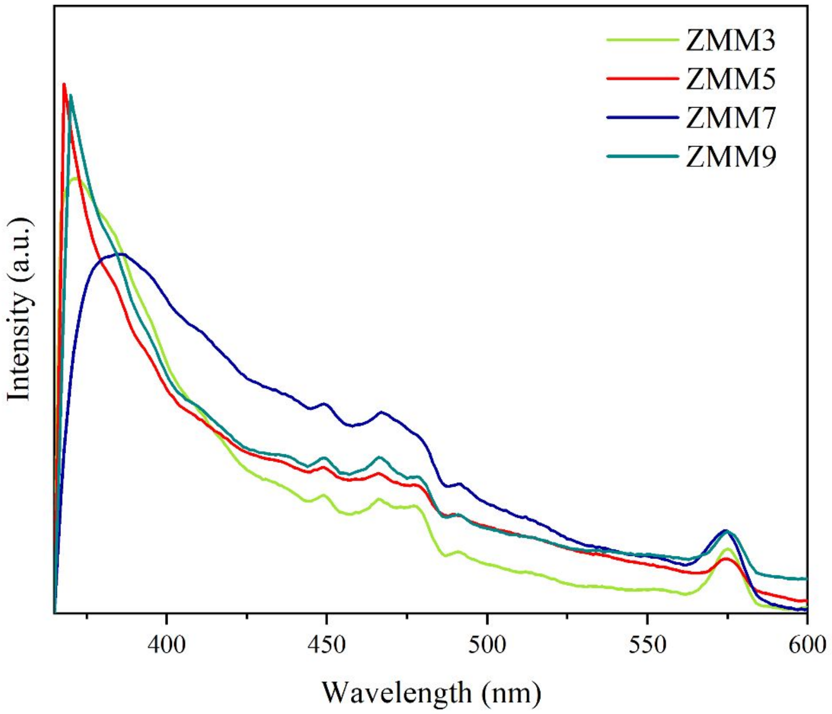


**Figure S2.** Photoluminescence spectra for the ZnO@$MnO_2$-Montmorillonite nanocomposite

## References

(1) Jerônimo, A. G.; Lins, A.; Barbosa, R.; Neves, L.; Albuquerque, W.; Trigueiro, P.; França, R.; Huaman, F. A.; Araujo, F. P.; Peña-Garcia, R. R. Ciprofloxacin Degradation via Advanced Oxidation Using La/Cu Co-Doped ZnO Photocatalysts. Mater Res Bull 2026, 193. https://doi.org/10.1016/j.materresbull.2025.113704.

(2) Wang, H.; Lu, Z.; Qian, D.; Li, Y.; Zhang, W. Single-Crystal α-MnO 2 Nanorods: Synthesis and Electrochemical Properties. Nanotechnology 2007, 18 (11), 115616. https://doi.org/10.1088/0957-4484/18/11/115616.

(3) Trigueiro, P.; Pereira, F. A. R.; Guillermin, D.; Rigaud, B.; Balme, S.; Janot, J.-M.; dos Santos, I. M. G.; Fonseca, M. G.; Walter, P.; Jaber, M. When Anthraquinone Dyes Meet Pillared Montmorillonite: Stability or Fading upon Exposure to Light? Dyes and Pigments 2018, 159 (June), 384–394. https://doi.org/10.1016/j.dyepig.2018.06.046.

(4) Albuquerque, W.; Trigueiro, P.; Silva, B. V; Neves, L.; Almeida, L. C.; Peña-Garcia, R. R. A Novel $RuO_2$@ZnO-Alginate-Halloysite Composite for the Effective Degradation of Eosin Yellow Dye and Ciprofloxacin Drug. Mater Res Bull 2024, 113178. https://doi.org/https://doi.org/10.1016/j.materresbull.2024.113178.

(5) França, R.; Araujo, F. P.; Castro-Lopes, S.; Neves, L.; Melo, A.; Jerônimo, A. G.; Osajima, J. A.; Guerra, Y.; Almeida, L. C.; Peña-Garcia, R. Effect of Cr Cations Addition on the Structural, Morphological, Optical, and Photocatalytic Properties of Er-Doped ZnO Structures. Mater Today Commun 2023, 37, 107419. https://doi.org/https://doi.org/10.1016/j.mtcomm.2023.107419.

(6) Soares, A. S.; Castro-Lopes, S.; Cabrera-Baez, M.; Milani, R.; Padrón-Hernández, E.; Farias, B. V.; Soares, J. M.; Gusmão, S. S.; Viana, B. C.; Guerra, Y.; Oliveira, C. S.; Peña-Garcia, R. The Role of PH on the Vibrational, Optical and Electronic Properties of the Zn FeO Compound Synthesized via Sol Gel Method. Solid State Sci 2022, 128 (April), 106880. https://doi.org/10.1016/j.solidstatesciences.2022.106880.

(7) Soares, A. S.; Castro-Lopes, S.; Cabrera-Baez, M.; Milani, R.; Padrón-Hernández, E.; Farias, B. V; Soares, J. M.; Gusmão, S. S.; Viana, B. C.; Guerra, Y.; Oliveira, C. S.; Peña-Garcia, R. The Role of PH on the Vibrational, Optical and Electronic Properties of the Zn1-XFexO Compound Synthesized via Sol Gel Method. Solid State Sci 2022, 128, 106880. https://doi.org/https://doi.org/10.1016/j.solidstatesciences.2022.106880.

(8) Castro-Lopes, S.; Guerra, Y.; Silva-Sousa, A.; Oliveira, D. M.; Gonçalves, L. A. P.; Franco, A.; Padrón-Hernández, E.; Peña-Garcia, R. Influence of PH on the Structural and Magnetic Properties of Fe-Doped ZnO Nanoparticles Synthesized by Sol Gel Method. Solid State Sci 2020, 109. https://doi.org/10.1016/j.solidstatesciences.2020.106438.

(9) Castro-Lopes, S.; Pereira, J. P.; Guerra, Y.; Trigueiro, P.; Garcia-Fornaris, I.; Govea-Alcaide, E.; Hernández, E. P.-; Peña-Garcia, R. R. Exploring the Structural and Optical Properties of Zn1-x-YCuxReyO Compound Obtained by Solid-State Reaction. Ceram Int 2025. https://doi.org/10.1016/j.ceramint.2025.02.001.
